# Cytochrome *c* Deficiency Differentially Affects the In Vivo Mitochondrial Electron Partitioning and Primary Metabolism Depending on the Photoperiod

**DOI:** 10.3390/plants10030444

**Published:** 2021-02-26

**Authors:** Igor Florez-Sarasa, Elina Welchen, Sofia Racca, Daniel H. Gonzalez, José G. Vallarino, Alisdair R. Fernie, Miquel Ribas-Carbo, Nestor Fernandez Del-Saz

**Affiliations:** 1 Centre for Research in Agricultural Genomics (CRAG) CSIC-IRTA-UAB-UB, Campus UAB Bellaterra, 08193 Barcelona, Spain; 2 Instituto de Agrobiotecnología del Litoral (CONICET-UNL), Cátedra de Biología Celular y Molecular, Facultad de Bioquímica y Ciencias Biológicas, Universidad Nacional del Litoral, Santa Fe 3000, Argentina; ewelchen@fbcb.unl.edu.ar (E.W.); sofia.racca@hotmail.com (S.R.); dhgonza@fbcb.unl.edu.ar (D.H.G.); 3 Max-Planck-Institute of Molecular Plant Physiology, Am Mühlenberg 1, 14476 Potsdam-Golm, Germany; Vallarino@mpimp-golm.mpg.de (J.G.V.); Fernie@mpimp-golm.mpg.de (A.R.F.); 4 Research Group on Plant Biology, Balearic Islands University, Ctra Valldemossa km 7.5, 07122 Palma de Mallorca, Spain; mribas@uib.cat; 5 Laboratorio de Fisiología Vegetal, Departamento de Botánica, Facultad de Ciencias Naturales y Oceanográficas, Universidad de Concepción, 4030000 Concepción, Chile

**Keywords:** alternative oxidase (AOX), cytochrome c (CYTc), oxygen isotope discrimination, metabolite profiling, photoperiod, primary metabolism

## Abstract

Plant respiration provides metabolic flexibility under changing environmental conditions by modulating the activity of the nonphosphorylating alternative pathways from the mitochondrial electron transport chain, which bypass the main energy-producing components of the cytochrome oxidase pathway (COP). While adjustments in leaf primary metabolism induced by changes in day length are well studied, possible differences in the in vivo contribution of the COP and the alternative oxidase pathway (AOP) between different photoperiods remain unknown. In our study, in vivo electron partitioning between AOP and COP and expression analysis of respiratory components, photosynthesis, and the levels of primary metabolites were studied in leaves of wild-type (WT) plants and cytochrome c (CYTc) mutants, with reduced levels of COP components, under short- and long-day photoperiods. Our results clearly show that differences in AOP and COP in vivo activities between WT and *cytc* mutants depend on the photoperiod likely due to energy and stress signaling constraints. Parallel responses observed between in vivo respiratory activities, TCA cycle intermediates, amino acids, and stress signaling metabolites indicate the coordination of different pathways of primary metabolism to support growth adaptation under different photoperiods.

## 1. Introduction

Respiration is a vital process for leaf primary metabolism because it provides ATP and carbon skeletons to sustain photosynthetic carbon (C) and nitrogen (N) metabolism required for plant growth and survival [1,2,3]. In addition, leaf mitochondrial respiration allows the dissipation of excess energy from chloroplasts in order to maintain cell redox balance [2,4,5]. Oxidative phosphorylation drives ATP synthesis in the mitochondrial electron transport chain (mETC) through an ATP synthase (or complex V) that is coupled with oxygen (O_2_) consumption mainly via the cytochrome c oxidase (COX) pathway (COP). This pathway is constituted by the ubiquinone (UQ) pool, complex III, cytochrome c (CYTc), and complex IV or COX [6,7]. CYTc mediates the connection of many metabolic routes, thus probably acting as a cell component that integrates energy, redox, and stress-related parameters into growth responses [8]. It plays important roles in programmed cell death [8,9,10], the import of proteins to the intermembrane space [11], the synthesis of ascorbic acid [12], and the detoxification of methylglyoxal and D-lactate [8,13,14]. Evidence from in vivo activity determinations denotes the vital importance of COP respiration as in the absence of stress it accounts for 50–90% of total respiration [5]. The remaining percentage in plants corresponds to the O_2_ consumption via an alternative nonphosphorylating electron transport pathway that competes with the COP for electrons of the UQ pool [15]. This is achieved due to the existence of the alternative oxidase (AOX) located in the inner mitochondrial membrane that, together with the UQ pool, composes the AOX pathway (AOP). Different roles of the AOP have been reported, including the maintenance of respiration and central metabolism pathways under COP restriction [5]. The inhibition of COP activity occurs under different stress conditions, including drought, low temperature, and nutrient limitation, as well as after exposure of plant tissues to allelochemicals, heavy metals, and nitric oxide [5]. Under such COP restriction, the mETC components become highly reduced, and the generation of reactive oxygen species (ROS) and nitrogen species (RNS) in the mitochondria increases with its consequences for protein oxidation and malfunction [16,17,18,19]. The AOP is thought to preserve COX function by limiting ROS and RNS generation that can lead to protein oxidative damage [3] and, at the same time, keeping the activity of (photo) respiratory metabolism essential for chloroplast redox and carbon balance [5].

The roles of mitochondrial respiration in photosynthetic tissues have been long debated [2,3,20,21,22]. As mentioned above, the AOP and other nonphosphorylating alternative pathways in mitochondria, such as the external (NDex) and internal (NDin) type II NAD(P)H dehydrogenases, contribute to cell redox and carbon balance as they function independently of the adenylate control [23,24]. Several pieces of evidence for these roles of the mitochondrial alternative pathways have come from studies using transgenic or mutant plants with altered levels of the alternative pathway components [3,5,24]. On the other hand, recent reports based on flux balance analysis and in vivo fluorescent sensors strongly support earlier classical studies suggesting the important role of mitochondria in providing ATP to the cytosol from photosynthetic cells, which can be used for the synthesis and export of sucrose in mature leaves or for growth in immature growing leaves [1,25]. As a further step, the use of genetically modified plants with altered components of the ATP-producing COP in combination with in vivo analysis could provide key experimental evidence for a better understanding of the impact of COP restrictions on leaf metabolism, thus avoiding side effects of chemical inhibitors’ application or complex effects due to environmental stress conditions. In this context, a considerable amount of literature has reported profound metabolic alterations and slow-growing phenotypes in mutants affected in different subunits of the complex I (CI) of the mETC [26,27,28,29]. A common effect observed in most CI mutants is the induction of the nonphosphorylating alternative pathways, mainly AOX, NDin, and/or NDex, at the transcript or protein levels. However, such inductions in CMSII tobacco and MSC16 cucumber plants did not correlate with increases in the in vivo AOP activity [26], which can be determined with a noninvasive oxygen isotope discrimination technique [30]. In turn, the lack of CI activity in tobacco CMSII plants was compensated by a higher in vivo COP activity [31,32,33] that resulted in higher or similar total leaf ATP pool [32,33,34,35]. On the other hand, increases in ROS production and in vivo AOP activity were observed in *Arabidopsis* CI mutants despite displaying similarities with the mentioned tobacco and cucumber mutants with respect to primary metabolism changes [28]. Indeed, Pétriacq et al. [28] reported and discussed that differences in metabolic and growth phenotypes observed in CI mutants depend on photoperiod conditions and probably on other environmental changes. In this respect, there is still a lack of information about the photoperiod effects on the in vivo activities of the AOP and COP and their particular responses in respiratory mutants.

It is well known that day length affects growth by controlling the daily assimilation of C, the proportion of the photosynthate accumulated as starch, the levels of organic acids, and the protein content [36,37]. In this context, differences in carbohydrate metabolism induced by day length are expected to correlate with changes in the COP respiratory rates, bearing in mind, as commented above, the importance of mitochondrial ATP for sucrose synthesis and export as well as for cell growth. Interestingly, CYTc, a crucial component of COP in plants [38], has been suggested to play a role in regulating plant growth, linking carbon utilization and hormonal pathways [7]. Increased sugar levels and starch synthesis in *cytc* mutants have been observed to be dependent on photoperiod [7], while the AOX capacity has been found to be increased in *cytc* mutants grown under long-day (LD) conditions. Such carbohydrate changes under LD have not been evident in CYTc overexpressors, which have displayed lower AOX capacity [7]. Metabolomic analysis and respiratory capacities have not been determined in *cytc* altered plants under both short-day (SD) and long-day (LD) photoperiods, and more importantly, their in vivo AOP and COP activities remain unknown under both photoperiods. Considering the lack of correlation between in vivo respiratory activities and their protein amounts and/or capacities observed in other respiratory mutants (i.e., CI mutants commented above), such in vivo AOP and COP determinations remain key to better understanding the link between mitochondrial energetics and cell primary metabolism under different photoperiods. 

In the present study, *Arabidopsis thaliana* Col-0 wild-type (WT) and *cytc* double mutant plants, with highly reduced levels of CYTc protein and a decrease in all subunits from complex IV [38], were grown under SD and LD photoperiods in order to test the consequence of COX restriction in different scenarios of energy and carbon demands. Oxygen isotope discrimination, Western blot, qPCR, gas exchange, chlorophyll fluorescence, and gas chromatography–mass spectrometry (GC–MS) analysis were applied to leaves of WT and *cytc* mutant plants to determine the in vivo activities of the AOX and COX pathways, the protein and transcript levels of representative components of these pathways, and the photosynthesis and levels of primary metabolites. The hypotheses behind this research were that (i) an adjustment of sugar metabolism and downstream metabolic pathways to an increased photoperiod could be accompanied by higher rates of in vivo COP respiration; (ii) in *cytc* mutant plants, restrictions on in vivo COP activity could lead to an impaired central metabolism and growth depending on the photoperiod (i.e., energy-demanding conditions) and the response of the respiratory bypass via AOP; and (iii) the AOP response under COX restriction could help keep some respiration functions (i.e., photosynthesis and carbon skeleton provision). Our results indeed clearly show a different in vivo mitochondrial electron partitioning between AOP and COP in the *cytc* mutants, as compared with WT, which depends on the photoperiod. These differences in the in vivo AOP and COP responses are discussed in the context of the previously reported growth inhibition of the *cytc* mutants together with the expression changes and reconfiguration of the primary metabolism observed here.

## 2. Results

### 2.1. Differences in the Relative Expression of Respiratory Chain Components between Cytc Mutants and WT Plants under Short- and Long-Day Photoperiods

The *cytc* mutants grown under short-day (SD) and long-day (LD) photoperiods displayed a delay in rosette development (Figure S1A), and therefore, all analyses were performed in leaves from plants at the same developmental stage (i.e., the same number of rosettes leaves). This means that there was approximately 1 week of difference in the ages (measured as number of days after sowing) of WT and *cytc* mutant plants (see Materials and Methods for details). Western blot (Figure S1B) and qPCR (Figure S1C) analyses confirmed the deficiency in CYTc in both mutant lines grown under both photoperiods. 

**Figure 1:**
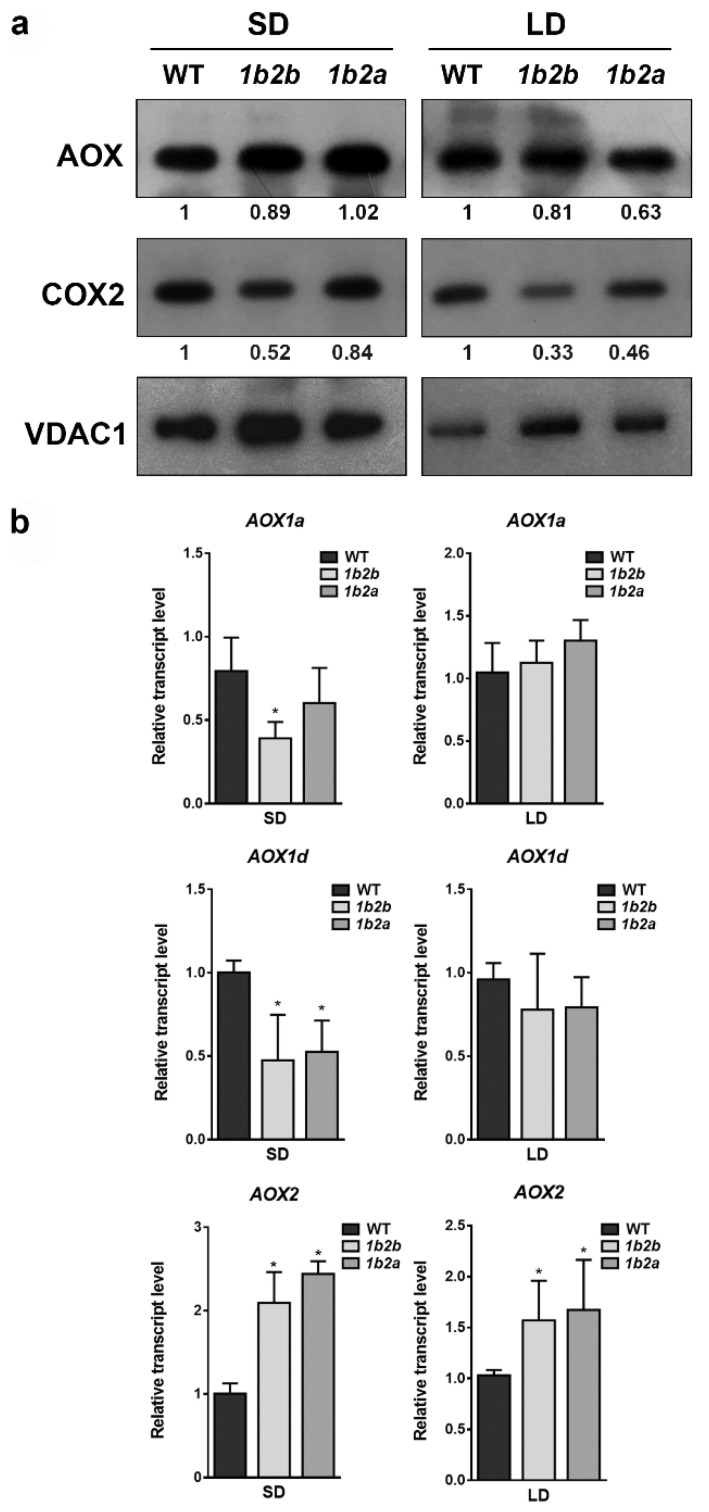
AOX and COX2 levels in leaves of wild-type (WT) and *cytc* mutants (*1b2a* and *1b2b*) grown in short-day (SD) and long-day (LD) photoperiods. (**a**) Western blot analysis of AOX and COX2 protein levels from total leaf protein extracts. The intensities of the signals from AOX and COX2 were normalized to those from porin (VDAC) and are expressed relative to WT levels. The normalized intensity values shown are averages of two independent blots. (**b**) Transcript levels for *AOX1a*, *AOX1d*, and *AOX2* genes evaluated in the same plants as in (**a**). Bars represent the means ± SD of three biological replicates. Asterisks indicate significant differences (*p* < 0.05) with the WT plants of the same stage according to LSD Fisher tests.

Total AOX and COX2 proteins were immunodetected together with the mitochondrial porin (VDAC) in order to compare the relative levels of mitochondrial proteins in WT and *cytc* mutants grown under both photoperiods (Figure 1a). The AOX protein level was similar between WT and *cytc* mutants under both SD and LD conditions. On the other hand, the levels of COX2 were lower in *cytc* mutants as compared with WT under both photoperiods. The reduction of COX2 levels was more pronounced under LD (more than 50% reduction in both lines) than under SD conditions.

The relative levels of transcripts encoding the AOX protein were also compared between WT and *cytc* mutants grown under both photoperiods (Figure 1b). Under SD, the transcript levels of *AOX1a* (only in *1a2b* line) and *AOX1d* were significantly lower in *cytc* mutants than in WT. On the contrary, the transcript levels of *AOX2* were higher in *cytc* mutants than in WT grown under SD. Under LD, the transcript levels of *AOX2* were also higher in *cytc* mutants, while the levels of *AOX1a* and *AOX1d* were not significantly different from WT. 

### 2.2. Respiration, Electron Partitioning to the AOX Pathway, and Photosynthesis in Cytc Mutants and WT Plants under Short- and Long-Day Photoperiods

Preliminary experiments were performed in order to test whether respiration rates and the electron partitioning to the AOP (τ_a_) were variable along the daytime. We compared rates of total oxygen consumption (V_t_) and τ_a_ between WT and *cytc* mutants under both photoperiod conditions (SD and LD) at different daytime ranges of 2 hours (i.e., approximately the measurement time for the respiration and oxygen isotope discrimination analysis). No significant effects of measurement time on the two respiratory parameters were detected at any photoperiod or genotype when analyzing differences to the first daytime measurements (Figure S2; *p* < 0.05; one-way ANOVA).

There was a significant effect of photoperiod on total oxygen uptake (V_t_) and on the in vivo activities of the cytochrome (*v*_cyt_) and the alternative oxidase (*v*_alt_) pathways (Table 1).

**Figure 2:**
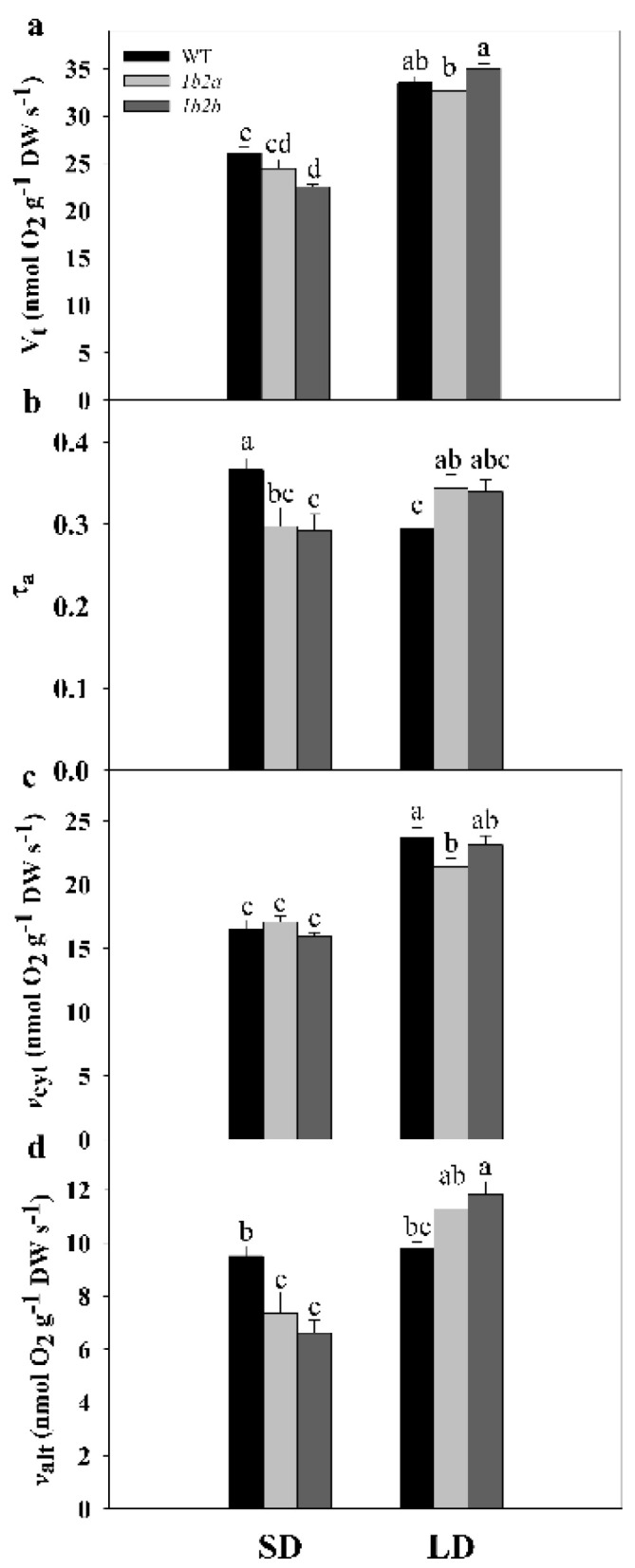
Respiration and electron partitioning between cytochrome and alternative pathways in leaves of wild-type (WT) and *cytc* mutants (*1b2a* and *1b2b*) grown in short-day (SD) and long-day (LD) photoperiods. (**a**) Total respiration (V_t_), (**b**) electron partitioning to the AOX pathway (τ_a_), (**c**) COX pathway activity (*v*_cyt_), and (**d**) AOX pathway activity (*v*_alt_). Values are means ± SE of six to nine replicates. Significant differences are denoted by different letters (*p* < 0.05; two-way ANOVA).

In WT plants, V_t_ and *v*_cyt_ were significantly higher by 29% and 43%, respectively, under LD compared with SD, while *v*_alt_ remained similar (Figure 2). In *cytc 1b2a* plants, V_t_, *v*_cyt_, and *v*_alt_ were significantly higher by 34%, 25%, and 53%, respectively, under LD compared with SD, whereas in *cytc 1b2b* plants, these parameters were significantly higher by 55%, 45%, and 79%. In addition, there was a significant effect of the interaction of the two factors (photoperiod × genotype) on all respiratory parameters (Table 1). Notably, a different pattern of τ_a_ was detected in WT and *cytc* mutants when SD and LD conditions were compared. The τ_a_ was significantly lower (ca. 20%) in WT plants under LD when compared with SD, while it was higher (ca. 16%) in the two *cytc* mutants. Among *cytc* mutants, V_t_ was significantly lower by 14% only in *cytc 1b2b* when compared with WT plants under SD, while *cytc 1b2a* displayed an intermediate V_t_ (Figure 2). In both *cytc* mutants grown under SD conditions, τ_a_ was significantly lower by 20% when compared with WT plants, because *v*_alt_ was significantly lower by 26% in the two genotypes. Under LD conditions, τ_a_ was higher by 17% in both *cytc* mutants. In this case, τ_a_ changes were due to the increases in *v*_alt_ together with the decreases in *v*_cyt_ displayed by *cytc* mutants. In particular, *v*_cyt_ was significantly lower by 10% in *cytc 1b2a* plants in comparison with WT plants, while *cytc 1b2b* displayed intermediate *v*_cyt_. On the other hand, *v*_alt_ was significantly higher by 17% in *cytc 1b2b* when compared with WT, while *cytc 1b2a* displayed intermediate *v*_alt_.

In order to further investigate the role of the mitochondrial electron transport chain in the response of plants to different photoperiods, measurements of net photosynthesis (A_N_), stomatal conductance (g_s_), and photosynthetic electron transport rate (ETR) were performed in *cytc* mutants and WT plants under SD conditions. No significant differences in A_N_, g_s_, or ETR were found among genotypes (*p* > 0.05; one-way ANOVA) (Table S1). Under LD conditions, similar values of ETR were found among genotypes. There was no significant effect of photoperiod, genotype, and their interaction on the ETR (*p* > 0.05; two-way ANOVA).

### 2.3. Metabolite Profiling in Cytc Mutants and WT Plants under Short- and Long-Day Photoperiods

In order to further investigate the metabolic changes underlying the responses of in vivo respiration and photosynthesis in the *cytc* mutants, gas chromatography–time-of-flight mass spectrometry (GC–TOF–MS) metabolite profiling analysis was performed on rosette samples from plants grown under SD and LD conditions (Table 2). A total of 49 metabolites were annotated after GC–TOF–MS analyses (Table S2). In general, differences to WT displayed by the *cytc* mutants were consistent in both lines, particularly under SD, with few exceptions (glycine, tryptophan, tyrosine, galactinol, and putrescine). Under LD, more metabolites were added to these exceptions (alanine, asparagine, glutamine, ornithine, citrate, phosphate, succinate, fructose, glucose, G6P, maltose, raffinose, sucrose, GABA, and beta-alanine), perhaps indicating some pleiotropic effects due to differential characteristics of the T-DNA lines (Table 2).

**Figure 3:**
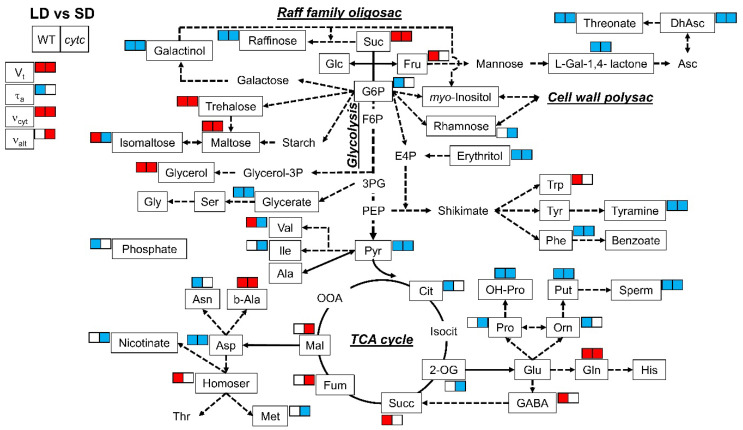
Metabolic model showing comparisons of leaf primary metabolites and respiratory parameters between LD and SD in WT and *cytc* mutants. Colored metabolites are those showing to be significantly (*p* < 0.05) higher (red) or lower (blue), determined by Duncan’s test. For *cytc* mutants, highlighted metabolites are those displaying consistent changes between photoperiods in both mutant lines. Data used were taken from Figure 2 and Table 2. Suc, sucrose; Glc, glucose; Fru, fructose; G6P, glucose-6-phosphate; F6P, fructose-6-phosphate; E4P, erythrose-4-phosphate; 3PG, 3-phosphoglycerate; PEP, phosphoenolpyruvate; Pyr, pyruvate; Cit, citrate; Isocit, isocitrate; 2-OG, 2-oxoglutarate; Succ, succinate; Fum, fumarate; Mal, malate; OOA, oxaloacetate; Asp, aspartate; Asn, asparagine; Thr, threonine; b-Ala, beta-alanine; Homoser, homoserine; Met, methionine; Val, valine; Ile, isoleucine; Ala, alanine; Ser, serine; Gly, glycine; Glycerol-3P, glycerol-3-phosphate; Glu, glutamate; Gln, glutamine; His, histidine; GABA, gamma-aminobutyrate; Pro, proline; OH-Pro, 4-hydroxy-proline; Orn, ornithine; Put, putrescine; Sperm, Spermine; Trp, tryptophan; Tyr, tyrosine; Phe, phenylalanine; L-Gal-1,4-lactone, L-galactono-1,4-lactone; Asc, ascorbate; DhAsc, dehydroascorbate.

All lines consistently displayed differences in several metabolites between SD and LD conditions (Table 2 and Figure 3). With regard to sugars and sugar alcohols, the levels of sucrose, trehalose, maltose, and glycerol were higher in LD than in SD plants. On the contrary, raffinose, galactinol, and erythritol were lower in LD plants. With regard to organic acids and derivatives, all plants grown in LD displayed lower levels of glycerate, pyruvate, dehydroascorbate, threonate, and L-galactono-1,4-lactone as compared with SD-grown plants. Some amino acids and derivatives displayed common SD vs. LD differences in all genotypes, including decreased levels of phenylalanine, aspartate, putrescine, spermidine, hydroxy-proline, and tyramine as well as increased levels of glutamine and beta-alanine. On the other hand, the levels of other metabolites in SD-grown plants were different from those in LD exclusively in *cytc* mutant plants; isomaltose, rhamnose, nicotinate, and 2-oxoglutarate, as well as valine, isoleucine, proline, and methionine, were lower in LD than in SD conditions only in *cytc* mutants. In addition, fumarate and malate were higher in LD conditions only in *cytc* mutants. From another perspective, glucose-6-phosphate, citrate, phosphate, ornithine, and asparagine were lower in LD than in SD conditions only in WT plants, while fructose, succinate, GABA, tryptophan, and homoserine were higher.

**Figure 4:**
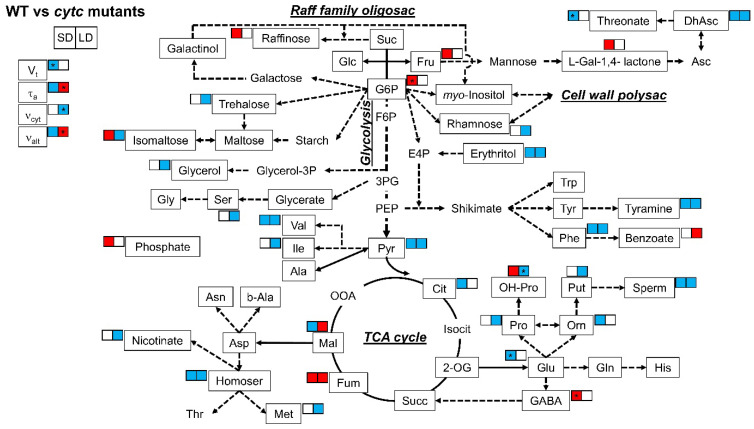
Metabolic model showing comparisons of leaf primary metabolites and respiratory parameters between WT and *cytc* mutants under SD and LD. Colored metabolites are those showing to be significantly (*p* < 0.05) higher (red) or lower (blue), determined by Duncan’s test. For *cytc* mutants, highlighted metabolites are those displaying consistent changes in both mutant lines as compared with WT in each photoperiod. Data used were taken from Figure 2 and Table 2. See Figure 3 for the abbreviations. * Only one line significantly (*p* < 0.05) different from WT but the other line with the same trend and intermediate response.

When differences between WT and *cytc* mutants were analyzed (Table 2 and Figure 4), some metabolites consistently displayed altered patterns in the *cytc* mutants compared with WT under both SD and LD conditions. Dehydroascorbate, erythritol, tyramine, phenylalanine, spermidine, homoserine, pyruvate, and serine were consistently lower in *cytc* mutants, while only fumarate was consistently higher under both SD and LD conditions. On the other hand, several metabolites displayed specific changes in *cytc* mutants. Notably, isomaltose and malate displayed opposite differences between WT and *cytc* mutants depending on the photoperiod conditions (also hydroxyl-proline showed a similar trend). Fructose, raffinose, L-galactono-1,4-lactone, phosphate, and hydroxyl-proline were higher in both *cytc* mutants only under SD (also glucose-6-phosphate and GABA showed similar trends), while benzoate was higher only under LD. Rhamnose, glycerol, serine, isoleucine, nicotinate, methionine, proline, and putrescine were only lower in *cytc* mutants than in WT under LD conditions, while ornithine and citrate were only lower under SD (also 2-oxoglutarate and threonate showed similar trends).

## 3. Discussion

Sugar signaling mechanisms were suggested to be involved in the control of sucrose partitioning into respiratory pathways [37,39,40]. However, the in vivo contribution of the two respiratory pathways of the mitochondrial electron transport chain under different photoperiods remained unknown. In the present research, we evaluated, for the first time, photoperiod-dependent differences in the in vivo activities of the AOP and COP in *Arabidopsis* plants. By using *cytc* mutant plants, we also tested whether reduced levels of CYTc could induce alterations in COP in vivo activity and whether this leads to a respiratory bypass via AOP for the benefit of primary metabolism and growth depending on the photoperiod conditions. 

Minor diurnal variations in leaf dark respiration of plants grown under controlled chamber conditions have been previously reported in Columbia (Col-0) WT *Arabidopsis* [41,42,43] and cucumber plants [44]. Correspondingly, respiration rates measured in our study were generally similar along the daytime in all genotypes under both SD and LD (Figure S2). Despite the marked diurnal regulation of transcript levels from central metabolism enzymes, their extractable activities do not generally display diurnal changes [45]. On the other hand, several enzyme activities involved in glycolysis and respiration displayed pronounced decreases in short as compared with long photoperiods [36]. Nevertheless, tissue extractable activities from respiratory enzymes do not necessarily indicate their activities in vivo, which are frequently lower and post-translationally regulated [46]. In our study, we observed that the higher total respiration under LD as compared with SD in all lines was mainly due to a higher in vivo COP activity (Figure 2). This is in agreement with the reported importance of mitochondrial ATP for sugar metabolism and plant growth [1,2,25]. Plants adapt to the photoperiod by adjusting growth rate according to the availability of energy. Accordingly, faster leaf growth occurs under LD conditions [47]. *Cytc* mutants also exhibited higher rosette growth rate under LD than under SD conditions (until reproductive stage), but notably, mutants displayed delayed growth when compared with WT in both photoperiods [7]. We therefore analyzed photosynthesis as the other main process, together with respiration, determining plant growth and carbon balance, and we observed similar photosynthetic rates in WT and *cytc* mutants and under both photoperiods (Table S1). Our results, together with previous observations [38], denote that *cytc* mutants are able to modulate in vivo COP activity in response to photoperiod, thus allowing photosynthesis and increased growth under LD. Similar photosynthesis rates during longer photoperiod lead to higher accumulation of carbon as starch reserves, and therefore, more carbon reserves are accumulated to achieve higher relative growth rates [37]. Growth delays in mutants, therefore, suggest that CYTc deficiency affects carbon use efficiency. 

Carbohydrates are considered the main substrates used as energy and carbon source for the biosynthesis of the cell wall, proteins, and lipids. Increases in protein synthesis and carbohydrate levels were reported under long photoperiods [36]. Accordingly, all the lines in our study showed, at the beginning of the day, higher levels of sucrose, trehalose, and maltose (also glycerol) under LD as compared with SD (Table 2, Figure 3). These sugar changes are indicative of a decreased rate of starch synthesis in favor of sucrose synthesis and export as has been previously observed under longer photoperiods [36,37,48]. On the contrary, raffinose, galactinol, and erythritol were lower in LD plants, maybe as a consequence of the increased flux towards sucrose synthesis and its use for respiration (Figure 3). The higher respiration rates observed in all lines under LD (Figure 2) were accompanied by lower levels of the glycolytic product pyruvate and linked tricarboxylic acid (TCA) cycle intermediates, such as citrate and 2-oxoglutarate, depending on the genotypes, while downstream intermediates (succinate, fumarate, and malate) remained similar or higher (Figure 3). While these metabolite patterns likely represent higher TCA cycle activity in all lines under LD, important differences were detected in WT and *cytc* mutant plants. Fumarate and malate were significantly higher in LD than in SD only in *cytc* mutants, while the opposite was observed for 2-oxoglutarate (Figure 3). Moreover, malate and fumarate displayed higher levels under LD in *cytc* mutants when directly compared with WT (Figure 4). Although these metabolite changes only allow for speculation concerning alterations in carbon fluxes, we propose that the TCA cycle activity, operating in noncyclic flux mode under illumination [49], could be restricted in the mutants particularly under LD; thus, *cytc* mutants could have altered carbon flux from 2-oxoglutarate for supporting amino acid synthesis on one side and a restricted contribution to redox balance and aspartate synthesis from malate/fumarate on the other side of the TCA cycle working in noncyclic mode. Interestingly, we previously found significant positive and negative correlations between changes in fumarate and 2-oxoglutarate, respectively, with in vivo AOP activity under higher light, which suggests a link between the AOP and the TCA cycle operating in a noncyclic flux mode under stress [50]. Correspondingly, the electron partitioning to the AOP (τ_a_) was higher in *cytc* mutants and WT under LD (Figure 2 and Figure 4). The higher τ_a_ value displayed by the *cytc* mutants was explained by both significantly higher AOP and lower COP in vivo activities. Another interesting observation in our study is that succinate and GABA were higher under LD than under SD only in WT plants, thus indicating an altered GABA shunt in the *cytc* mutants. The GABA shunt is an integral part of the TCA cycle [51]. It provides an alternative carbon supply of succinate bypassing the 2-oxoglutarate dehydrogenase complex (OGDC) and succinyl Co-A synthetase catalyzed steps and also lowers the capacity for cyclic operation of the TCA cycle to provide reductants for respiration, which can be beneficial under stress [51,52]. A lower 2-oxoglutarate level under LD compared with SD was only observed in the *cytc* mutants and could indicate a higher activation of the GABA shunt that, in parallel with the in vivo AOX activation, could respond as a redox bypass system. Curiously, we have previously observed parallel changes in the GABA shunt and in vivo AOX under nonstress [53] and stress conditions [50]. Although redox links can be suggested between these two alternative bypass mitochondrial pathways, the functional relationships between the AOP and the GABA shunt deserve more research in the future. We then propose that CYTc deficiency induces a pronounced reduction of COX2 levels (Figure 1), which restricts the in vivo COP activity under the higher respiratory energy and carbon demands imposed by the LD photoperiod (Figure 2). Although apparently minor, the observed reduction in the ATP-producing pathway sustained in time could significantly have an impact on plant growth and development. Under these conditions, the higher reduction levels of the UQ pool in the *cytc* mutants could be alleviated by an induction of the in vivo AOP activity (Figure 2). This increased AOP activity could then diminish mitochondrial ROS production from a highly reduced electron transport chain while supporting TCA cycle functions related to photosynthesis and carbon skeleton provision for amino acid synthesis [3,5] under LD at the expense of reducing ATP yield. In this line, it is worth mentioning that phosphate levels were lower under LD as compared with SD only in WT (Table 2 and Figure 3), probably reflecting higher ATP turnover than in the *cytc* mutants. 

In parallel to the observed adjustments of respiratory metabolism, the levels of several amino acids and derivatives were significantly lower in *cytc* mutants, particularly under LD (Figure 4). Whether these changes in amino acids affect protein synthesis (i.e., due to energy restriction in the mutants) remains elusive, but protein content is unlikely to be directly correlated with the abundance of amino acids [36]. This is because an increase in protein content may increase construction costs as the assimilation of inorganic nitrogen into amino acids and the subsequent conversion of amino acids to protein are energetically expensive processes [36,54,55,56]. In our study, restricted malate and fumarate metabolism under LD observed in the *cytc* mutants could be related to lower levels of aspartate-family amino acids, such as homoserine, methionine, and isoleucine (Figure 4), which are tightly connected to TCA cycle activity [57]. On the other hand, lower erythritol in concert with lower phenylalanine and tyramine levels (Figure 4) could indicate a decreased flux through the shikimate pathway and derived amino acids. While altered TCA cycle regulation has previously been reported to affect secondary metabolism, including flavonoid biosynthesis [58,59], consequences of an altered shikimate pathway in *cytc* mutants deserve further investigation. Shikimate was tightly correlated with total amino acid levels under different photoperiods and was proposed as a read-out for amino acid biosynthesis [37]. Moreover, the shikimate pathway flux has been estimated to consume more than 30% of the total carbon fixed by plants, particularly for the synthesis of phenylalanine [60,61]. Therefore, we propose that the restrictions in respiratory carbon and energy metabolism observed in *cytc* mutants likely affected amino acid and protein synthesis particularly under LD.

While metabolic and growth patterns observed in the *cytc* mutants under LD can be well explained by the alterations in protein levels and in vivo electron partitioning, the observed changes in respiratory metabolism under SD are perhaps less intuitive. Total respiration was lower in *cytc* mutants than in WT plants, which surprisingly was mainly due to a lower in vivo AOP activity (Figure 2). Apparently, a more efficient respiration in terms of ATP production should be beneficial for growth, although the in vivo activity of the ATP-producing COP remained unaltered in *cytc* mutants (Figure 2), likely related to a smaller reduction of COX2 levels under SD (Figure 1). The relationship between growth and AOP activity remains a matter of debate [3,62,63,64]. Under stress conditions, the beneficial effects of the AOP on photosynthesis are thought to outweigh growth potential losses due to the lower respiratory energy yield [3,64]. Under nonstress conditions, the AOP significantly contributes to respiration, and it is thought to also be important for the balance of carbon and energy metabolism under different developmental processes [5]. However, little experimental evidence exists regarding the role of the AOP under nonstress conditions. Suppression of AOX1 expression has not resulted in any reported decrease of the AOP in vivo activity under nonstress conditions, a desirable trait if we seek to understand the role of the AOP [5]. To the best of our knowledge, this is the first report of a respiratory mutant displaying a specific decrease in AOP in vivo activity under nonstress conditions. Notably, this was observed in parallel to different metabolic changes, which may indicate an altered stress signaling response. In line with this, *cytc* mutants displayed increased levels of stress-related metabolites, such as some ascorbate-related metabolites, GABA, 4-hydroxy-proline, and raffinose. Increased level of L-galactono-1,4-lactone together with lower levels of dehydroascorbate and threonate under SD (Figure 4) could be explained by decreases in GLDH activity as previously observed in the *cytc* mutants [38]. CYTc is a key component of mitochondrial ascorbate synthesis linking L-galactono-1,4-lactone oxidation and mETC activity potentially coordinating ascorbate synthesis [8,12,65]. Changes in these ascorbate-related metabolites could be linked to the observed reduction in AOP activity in the *cytc* mutants since a direct relationship between AOX expression and ascorbate levels was previously established in AOX-modified plants [66]. Changes in ascorbate-related metabolites and AOP activity could induce stress signaling responses affecting stress-related gene expression in other cellular compartments beyond mitochondria, as previously reported [8,38]. One possibility is that increased expression of *AOX2* in the *cytc* mutants (Figure 1b) is related to chloroplast redox balance since this isoform has been shown to be dually targeted to chloroplasts and mitochondria. However, changes in *AOX2* transcript levels were also observed under LD, while AOX1-type isoforms were only affected under SD, thus suggesting different regulatory pathways. The lower levels of *AOX1* transcripts in the *cytc* mutants (Figure 1b) support an altered retrograde signaling probably via mitochondrial ROS signals, as previously reported [3]. However, it is unlikely that the reduction of in vivo AOX activity was due to a restriction in the pathway capacity since AOX protein levels remained similar in all genotypes (Figure 1a). Notably, the lower levels of pyruvate, an AOX activator [67,68], may explain the decrease in activity of the AOP. In addition to pyruvate, reduced levels of malate and citrate, which can be linked to a decrease in the AOX activator oxaloacetate [68], were observed specifically under SD.

## 4. Conclusions

We here provided the first evidence of the in vivo activities of COX and AOX and their metabolic connections with primary metabolites under different photoperiods. Our results indicated that LD growth leads to higher rates of in vivo COP respiration together with an adjustment of sugar metabolism and downstream metabolic pathways promoting protein synthesis. Reduced levels of CYTc protein differently affected the energy efficiency of respiration depending on the photoperiod. CYTc deficiency induced reductions in COX2 protein levels that probably restricted energy metabolism via COP respiration under LD. Under these conditions, increased AOP in vivo activity likely aided the photosynthesis and growth responses, although with an altered TCA cycle and a restricted amino acid metabolism. By contrast, *cytc* mutants under SD displayed a specific decrease in AOP in vivo activity in parallel to different metabolic changes that suggested an altered stress signaling response. The metabolic interdependence between the GABA shunt, hormonal changes, and the AOP in modulating stress signaling under both stress and nonstress conditions deserves further investigation. 

## 5. Materials and Methods 

### 5.1. Plant Material and Growth Conditions 

Plants used in this study were wild-type (WT) Columbia-0 (Col-0) and double mutants in *CYTC-1* and *CYTC-2*, named *cytc 1b2a* and *cytc 1b2b*, as previously described [38]. WT plants and *cytc* mutants were grown under short-day (8/16 h light/dark) or long-day (16/8 h light/dark) photoperiods and controlled conditions of temperature (24 °C), relative humidity (above 50%), and light intensity (80 µmol m^−2^ s^−1^). WT plants and *cytc* mutants were grown until reaching similar developmental stages (before flowering, see Figure S1A) according to the previously reported developmental differences in leaf number and flowering time (Figure 3 in [7], i.e., 1 and 2 weeks of developmental delay in the mutants under SD and LD, respectively): for SD-grown plants, 6–7 and 7–8 weeks in WT and *cytc* mutant plants, respectively; for LD-grown plants, 3 and 4 weeks in WT and *cytc* mutant plants, respectively. Samples for leaf protein, transcript, and metabolite analyses were harvested at the beginning of the day (1 h after the onset of the light period). Photosynthetic and respiration analyses were performed at different daytimes, and they were not significantly affected by daytime as detailed below.

### 5.2. Protein and Transcript Levels

Protein levels were determined after Western blot analysis. Total protein extracts were prepared according to previous studies [69], separated on 10–16% tricine–SDS-PAGE [70], and transferred to PVDF membranes (GE Healthcare). Transfer was checked by Ponceau S staining. Blots were destained with several quick washes in distilled water and one wash in 1X TBS (10 mM Tris-HCl, pH 7.5, 150 mM NaCl), blocked for 1 h in 5% low-fat milk in TBST (1X TBS, 0,1% Tween 20), probed with specific antibodies (Table S3), and developed with anti-rabbit conjugated with horseradish peroxidase using the Agrisera ECL kit (AS16 ECL-S-N).

Transcript levels were analyzed after reverse transcription followed by quantitative PCR (RT-qPCR) analysis. RNA samples were prepared with TRIzol reagent (Thermo Fisher), followed by an additional step of LiCl precipitation. RT-qPCR analysis was performed according to previous studies [71]. First-strand cDNA synthesis was performed using the oligo(dT)18 primer and M-MLV reverse transcriptase (Promega) under standard conditions. qPCR was performed on an aliquot of the cDNA synthesis reaction with specific primers (Table S4) in an Applied Biosystems StepOne apparatus in a 20 µL final volume using 1 µL SYBR Green, 10 pmol of each primer, 3 mM MgCl2, dilutions of the reverse transcription reaction, and 0.2 µL Phire II polymerase (Thermo Scientific). Relative transcript levels were calculated by a comparative Ct method. Expression values were normalized using *PP2AA3* or *ACT2* and *ACT8* transcript levels as standards [72,73].

### 5.3. Respiration and Oxygen Isotope Discrimination Measurements 

Measurements of oxygen consumption and isotope discrimination during respiration were performed at 25 °C as previously described in order to determine the in vivo activities of the COX and AOX pathways in *Arabidopsis* leaves [62]. As for the end-point discrimination values, the oxygen isotope discrimination of the AOX pathway was determined after incubation with 10 mM KCN as described previously [62], and a mean value of 30.5‰ was used from all the measurements performed both in the mutant and wild-type plants because no differences were observed between lines. On the other hand, an end-point value of 20.9% corresponding to the oxygen isotope discrimination of the COX pathway was taken from previous measurements in *Arabidopsis* leaves [62]. Calculations of the electron partitioning and the activities of the AOX and COX pathways were performed as described previously [74]. As a preliminary experiment, V_t_ and τ_a_ were determined at three different daytime periods to test their variation along the daytime. For this preliminary analysis, we pooled data of the two mutant lines and evaluated whether values of V_t_ and τ_a_ in WT and *cytc* mutants corresponding to the second and third daytime periods differed from the first daytime period in each photoperiod condition (SD and LD) (Figure S2). Values are means ± SE for three to four and four to eight biological replicates for WT and *cytc* mutants, respectively, at each daytime period and photoperiod. For the experiments presented in Figure 2, six to nine replicates (each representing pools of leaves from at least two different plants) per line and photoperiod were performed for in vivo activities.

### 5.4. Leaf Gas Exchange and Chlorophyll Fluorescence Measurements 

In SD-grown plants, net CO_2_ assimilation (A_N_), stomatal conductance (g_s_), and chlorophyll fluorescence were measured simultaneously with an open infrared gas exchange analyzer system (Li-6400; Li-Cor Inc., Lincoln, NE, USA) equipped with a leaf chamber fluorometer (Li-6400–40, Li-Cor Inc.). Fully expanded leaves were clamped at the same daytimes for respiration analyses, and leaf chamber conditions were set to photosynthetically active photon flux density (PPFD) of 1000 µmol m^−2^ s^−1^ (provided by the light source of the Li-6400 with 10% blue light), 400 µmol CO_2_ mol^−1^ air (C_a_), and a temperature of 25 °C. After approximately 20 min, steady-state gas exchange and chlorophyll fluorescence measurements were performed in the light. The actual quantum efficiency of the photosystem II (PSII)-driven electron transport (PhiPSII) and the electron transport rate (ETR) were determined as previously described [75]. Five to eight measurements were performed in leaves from different SD-grown plants. In LD plants, PhiPSII was obtained from chlorophyll fluorescence measurements on leaves from 12 different plants with a portable pulse amplitude modulation fluorometer (PAM-2000, Walz, Effeltrich, Germany), and ETR was calculated as described previously [75].

### 5.5. Metabolite Profiling

Metabolite extractions were performed as described previously [76] using approximately 10 mg of lyophilized leaf tissue, previously frozen-powdered. Derivatization and GC–TOF–MS analyses were carried out as described previously [76]. Metabolites were identified manually by TagFinder software [77] using the reference library mass spectra and retention indices housed in the Golm Metabolome Database (http://gmd.mpimp-golm.mpg.de (accessed on 18 February 2021); [78]). The parameters used for the peak annotation of the 49 metabolites can be found in Table S2, which follows previously reported recommendations [79]. Data were normalized to the mean value of wild-type (WT) plants under SD conditions (i.e., the value of all metabolites for WT at SD was set to 1). Values presented are means ± SE of three to five replicates corresponding to rosette leaves from 6 to 10 different plants.

### 5.6. Statistical Analyses

A univariate analysis of variance (ANOVA) was performed to study the influence of photoperiod, genotype, and their interaction on each respiratory parameter or metabolite studied. The *p*-values of each factor (photoperiod, genotype, and interaction) for all parameters studied can be found in Table 1. A Duncan post hoc test was used to evaluate the significance of the differences between treatments and varieties (*p* < 0.05) in Figure 2 and Table 2. All these analyses were computed using the SPSS statistical software package, version 25 (IBM Corp., 2016, Armonk, New York, NY, USA).

## Figures and Tables

**Table 1:** Significance of sources of variation after two-way analysis of variance for each physiological parameter. The sources of variance were SD or LD conditions (photoperiod), plant genotype, and their interactions (photoperiod × genotype). V_t_, total respiration; τ_a_, electron partitioning to the AOX pathway; *v*_cyt_, COX pathway activity; *v*_alt_, AOX pathway activity; ETR, chloroplast electron transport rate. ns = no significant effect. * *p* < 0.05; ** *p* < 0.01; *** *p* < 0.001.

Parameter	Photoperiod	Genotype	Photoperiod × Genotype
**V_t_**	***	ns	**
**τ_a_**	ns	ns	***
***v*_cyt_**	***	ns	*
***v*_alt_**	***	ns	***
**ETR**	ns	ns	ns

**Table 2:** Relative metabolite levels in leaves of WT and *cytc* mutant plants grown under short-day (SD) and long-day (LD) photoperiods. Data are presented as means ± SE for three to five biological replicates normalized to the mean level of the WT plants under SD. Different letters denote significant differences (*p* < 0.05) between genotypes and photoperiods.

Metabolite		SD			LD	
	WT	*1b2a*	*1b2b*	WT	*1b2a*	*1b2b*
*Amino acids*						
Alanine	1 ± 0.23 ab	1.06 ± 0.02 ab	0.87 ± 0.03 b	1.24 ± 0.02 a	1.25 ± 0.03 a	0.74 ± 0.02 b
Asparagine	1 ± 0.02 a	1.02 ± 0.05 a	0.96 ± 0.06 a	0.6 ± 0.02 b	0.98 ± 0.03 a	0.69 ± 0.03 b
Aspartate	1 ± 0.09 a	1.1 ± 0.06 a	0.97 ± 0.09 a	0.42 ± 0.01 b	0.35 ± 0 b	0.38 ± 0.01 b
Glutamate	1 ± 0.04 ab	0.87 ± 0.03 bc	0.83 ± 0.06 c	1.03 ± 0.03 a	1.04 ± 0.01 a	0.92 ± 0.05 abc
Glutamine	1 ± 0.03 de	0.89 ± 0.03 e	1.12 ± 0.06 cd	1.27 ± 0.08 bc	1.98 ± 0.07 a	1.37 ± 0.08 b
Glycine	1 ± 0.02 a	0.95 ± 0.05 a	0.73 ± 0.03 b	1.05 ± 0.03 a	0.98 ± 0.02 a	0.62 ± 0.05 b
Histidine	1 ± 0.22	1.02 ± 0.09	0.8 ± 0.06	0.93 ± 0.05	0.76 ± 0.01	1 ± 0.05
Homoserine	1 ± 0.04 b	0.82 ± 0.05 c	0.8 ± 0.02 c	1.21 ± 0.05 a	0.95 ± 0 b	0.81 ± 0.01 c
Isoleucine	1 ± 0.05 a	0.91 ± 0.02 a	0.95 ± 0.04 a	0.99 ± 0.03 a	0.67 ± 0.03 b	0.64 ± 0.05 b
Methionine	1 ± 0.05 a	1.03 ± 0.04 a	0.91 ± 0.04 a	1.03 ± 0.06 a	0.27 ± 0.06 c	0.63 ± 0.05 b
Ornithine	1 ± 0.03 a	0.76 ± 0.02 b	0.75 ± 0.03 b	0.77 ± 0.02 b	0.48 ± 0.02 c	0.81 ± 0.05 b
Phenylalanine	1 ± 0.01 a	0.84 ± 0.04 b	0.85 ± 0.02 b	0.86 ± 0.05 b	0.67 ± 0 c	0.48 ± 0.02 d
Proline	1 ± 0.03 b	1.16 ± 0.06 a	1.08 ± 0.03 ab	0.97 ± 0.06 b	0.58 ± 0.01 c	0.56 ± 0.05 c
Tryptophan	1 ± 0.03 cd	0.89 ± 0.03 d	1.24 ± 0.12 bc	1.34 ± 0.08 b	0.74 ± 0.01 d	3.19 ± 0.14 a
Tyrosine	1 ± 0.02 ab	0.87 ± 0.05 b	1.06 ± 0.06 a	0.93 ± 0.06 ab	0.92 ± 0.01 ab	1.03 ± 0.04 a
Serine	1 ± 0.02 ab	0.95 ± 0.04 ab	1.01 ± 0.03 ab	1.04 ± 0.04 a	0.92 ± 0.01 bc	0.84 ± 0.02 c
Valine	1 ± 0.01 b	0.89 ± 0.01 c	0.86 ± 0.02 c	1.13 ± 0.06 a	0.67 ± 0.01 d	0.53 ± 0.02 e
*Organic acids*						
Benzoate	1 ± 0.07 bc	1.06 ± 0.04 bc	1.03 ± 0.05 bc	0.92 ± 0.06 c	1.2 ± 0.11 ab	1.27 ± 0.06 a
Citrate	1 ± 0.07 a	0.65 ± 0.03 b	0.77 ± 0.06 b	0.69 ± 0.03 b	0.71 ± 0.04 b	1 ± 0.07 a
Dehydroascorbate	1 ± 0.02 a	0.81 ± 0.05 b	0.73 ± 0.05 b	0.74 ± 0.03 b	0.4 ± 0 c	0.29 ± 0.01 c
Fumarate	1 ± 0.02 c	1.2 ± 0.04 b	1.2 ± 0.04 b	1.08 ± 0.03 bc	1.54 ± 0.11 a	1.46 ± 0.09 a
2-Oxoglutarate	1 ± 0.14 a	1.04 ± 0.16 a	0.91 ± 0.09 a	0.69 ± 0.08 ab	0.47 ± 0.03 b	0.48 ± 0.09 b
Glycerate	1 ± 0.03 a	1 ± 0.04 a	1.08 ± 0.03 a	0.83 ± 0.05 b	0.77 ± 0.01 b	0.88 ± 0.03 b
Malate	1 ± 0.06 c	0.8 ± 0.07 d	0.7 ± 0.04 d	0.97 ± 0.06 c	1.56 ± 0.02 a	1.23 ± 0.04 b
Nicotinate	1 ± 0.04 a	1.11 ± 0.08 a	1.11 ± 0.05 a	0.99 ± 0.05 a	0.76 ± 0.04 b	0.59 ± 0.02 c
Phosphate	1 ± 0.03 b	1.21 ± 0.08 a	1.2 ± 0.06 a	0.4 ± 0.03 c	0.55 ± 0 c	1.14 ± 0.03 ab
Pyruvate	1 ± 0.03 a	0.76 ± 0.04 b	0.71 ± 0.04 b	0.41 ± 0.02 c	0.22 ± 0.01 d	0.18 ± 0.01 d
Threonate	1 ± 0.05 a	0.94 ± 0.07 ab	0.83 ± 0.01 b	0.51 ± 0.02 c	0.45 ± 0.01 c	0.53 ± 0.02 c
Succinate	1 ± 0.08 c	0.98 ± 0.07 c	1.09 ± 0.01 c	1.48 ± 0.09 b	2.4 ± 0.03 a	1.13 ± 0.04 c
*Sugars and sugar alcohol*						
Erythritol	1 ± 0.03 a	0.85 ± 0.04 b	0.82 ± 0.03 b	0.8 ± 0.03 b	0.43 ± 0.01 c	0.38 ± 0.03 c
Fructose	1 ± 0.04 b	1.2 ± 0.05 a	1.25 ± 0.04 a	1.2 ± 0.06 a	1.26 ± 0.01 a	0.84 ± 0.03 c
Galactinol	1 ± 0.04 a	1.1 ± 0.04 a	0.86 ± 0.07 b	0.14 ± 0 d	0.12 ± 0 d	0.32 ± 0.02 c
Glucose	1 ± 0.02 b	1.01 ± 0.02 b	1.04 ± 0.03 ab	1.05 ± 0.02 ab	1.13 ± 0.08 a	0.9 ± 0.02 c
Glucose-6-P	1 ± 0.07 b	1.32 ± 0.12 a	1.08 ± 0.09 ab	0.52 ± 0.02 c	0.48 ± 0.01 c	1.02 ± 0.08 b
Glycerol	1 ± 0.06 c	0.93 ± 0.01 c	0.94 ± 0.03 c	1.8 ± 0.12 a	1.43 ± 0.14 b	1.41 ± 0.05 b
Myo-inositol	1 ± 0.03	1 ± 0.03	0.99 ± 0.02	0.87 ± 0.14	0.95 ± 0.01	0.84 ± 0.05
Isomaltose	1 ± 0.01 b	1.39 ± 0.05 a	1.44 ± 0.04 a	1.3 ± 0.03 a	0.47 ± 0.03 c	0.99 ± 0.08 b
maltose	1 ± 0.09 d	1.65 ± 0.08 c	1.13 ± 0.06 d	2.31 ± 0.17 b	2.96 ± 0.02 a	1.64 ± 0.04 c
Raffinose	1 ± 0.05 b	1.23 ± 0.11 a	1.41 ± 0.05 a	0.68 ± 0.06 c	0.26 ± 0 d	0.57 ± 0.06 c
Rhamnose	1 ± 0.06 a	1.06 ± 0.04 a	1.08 ± 0.04 a	1.05 ± 0.02 a	0.5 ± 0 b	0.48 ± 0.02 b
Sucrose	1 ± 0.07 c	0.9 ± 0.1 c	0.9 ± 0.09 c	2.65 ± 0.1 a	2.66 ± 0.02 a	2.08 ± 0.15 b
Trehalose	1 ± 0.04 cd	0.83 ± 0.04 d	0.85 ± 0.02 d	1.88 ± 0.09 a	1.39 ± 0.01 b	1.13 ± 0.08 c
*Other metabolites*						
GABA	1 ± 0.04 d	1.15 ± 0.03 cd	1.24 ± 0.07 c	1.54 ± 0.06 b	1.97 ± 0.1 a	1.07 ± 0.09 cd
Beta-alanine	1 ± 0.06 cd	1.06 ± 0.06 c	0.99 ± 0.01 c	1.3 ± 0.06 b	1.53 ± 0.02 a	1.22 ± 0.06 b
Galactono-1,4-lactone	1 ± 0.03 b	1.12 ± 0.05 a	1.19 ± 0.02 a	0.59 ± 0.01 c	0.52 ± 0.03 c	0.53 ± 0.02 c
4-Hydroxy-proline	1 ± 0.08 b	1.18 ± 0.07 a	1.17 ± 0.05 a	0.51 ± 0.02 c	0.43 ± 0.04 cd	0.33 ± 0.01 d
Putrescine	1 ± 0.06 a	0.98 ± 0.04 a	0.82 ± 0.02 b	0.77 ± 0.06 b	0.64 ± 0.02 c	0.47 ± 0.04 d
Spermidine	1 ± 0.03 a	0.81 ± 0.03 b	0.76 ± 0.02 b	0.65 ± 0.04 c	0.13 ± 0 d	0.13 ± 0 d
Tyramine	1 ± 0.04 a	0.87 ± 0.03 b	0.85 ± 0.02 b	0.85 ± 0.03 b	0.57 ± 0.02 c	0.43 ± 0.02 d

## Data Availability

Not applicable.

## Supplementary Materials

Supplementary materials can be found at https://www.mdpi.com/2223-7747/10/3/444/s1. Figure S1: (A) Representative images of WT and *cytc* mutant (*1b2a* and *1b2b*) plants grown under SD and LD photoperiod. (B) CYTc protein evels in total protein extracts from WT and *cytc* mutant plants. VDAC1 protein is shown as a mitochondrial loading control. (C) Transcript levels for *CYTc-1* and *CYTc-2* genes evaluated on these plants. The bars represent the mean±SD f thee biological replicates from each genotype. Asterisks indicate significant differences with WT plants (*p* < 0.01) according to t-tests. Figure S2: Results of total respiration (V_t_) and electron partitioning to the AOP in the leaves of WT plants and *cytc* mutants grown at SD (a,c) and LD (b,d) at three different daytime measurement periods (SD (a,c) 1–3 h, 4–6 h, 7–9 h and LD (b,d) 1–2 h, 3–5 h, 6–9 h). Values are means±SE for 3–4 and 4–8 biological replicates for WT and *cytc* mutants, respectively, at each photoperiod and daytime measurement periods. Table S1: Photosynthetic parameters in leaves of wild-type (WT) plants and cytc mutants (1b2a and 1b2b) under SD and LD conditions: net photosynthesis (AN), stomatal conductance (gs), and electron transport rate (ETR). Data represent means ± SE of 5–9 and 12 replicates in SD and LD conditions, respectively. Table S2: Overview of the metabolite reporting list.
