# Supplementary material for: Cytochrome c Deficiency Differentially Affects the In Vivo Mitochondrial Electron Partitioning and Primary Metabolism Depending on the Photoperiod
Source: Plants (Basel). 2021 Feb 26;10(3):444. doi: 10.3390/plants10030444 (PMC7996904; doi:10.3390/plants10030444)
Supplement: Supplementary file 1 [file plants-10-00444-s001.zip › Florez-Sarasa et al_Plants_Supplementary Material/Table S1.docx]

**Table S1.** Photosynthetic parameters in leaves of wild-type (WT) plants and *cytc* mutants (*1b2a* and *1b2b*) under SD and LD conditions: net photosynthesis (A_N_), stomatal conductance (g_s_), and electron transport rate (ETR). Data represent means ± SE of 5-9 and 12 replicates in SD and LD conditions, respectively.

| Parameter SD | WT | *1b2a* | *1b2b* |
| --- | --- | --- | --- |
| A_N_ (µmol m^-2^ s^-1^) | 7.45 ± 0.38 | 7.18 ± 0.51 | 6.84 ± 0.43 |
| g_s_ (mol m^-2^ s^-1^) | 0.101 ± 0.006 | 0.104 ± 0.011 | 0.088 ± 0.010 |
| ETR (µmol m^-2^ s^-1^) | 45.3 ± 2.0 | 44.5 ± 2.5 | 43.7 ± 1.8 |
| Parameter LD |  |  |  |
| ETR (µmol m^-2^ s^-1^) | 45.3 ± 1.5 | 46.7 ± 2.5 | 46.0 ± 1.7 |
