# Supplementary figures and images for: Cytochrome c Deficiency Differentially Affects the In Vivo Mitochondrial Electron Partitioning and Primary Metabolism Depending on the Photoperiod
Source: Plants (Basel). 2021 Feb 26;10(3):444. doi: 10.3390/plants10030444 (PMC7996904; doi:10.3390/plants10030444)

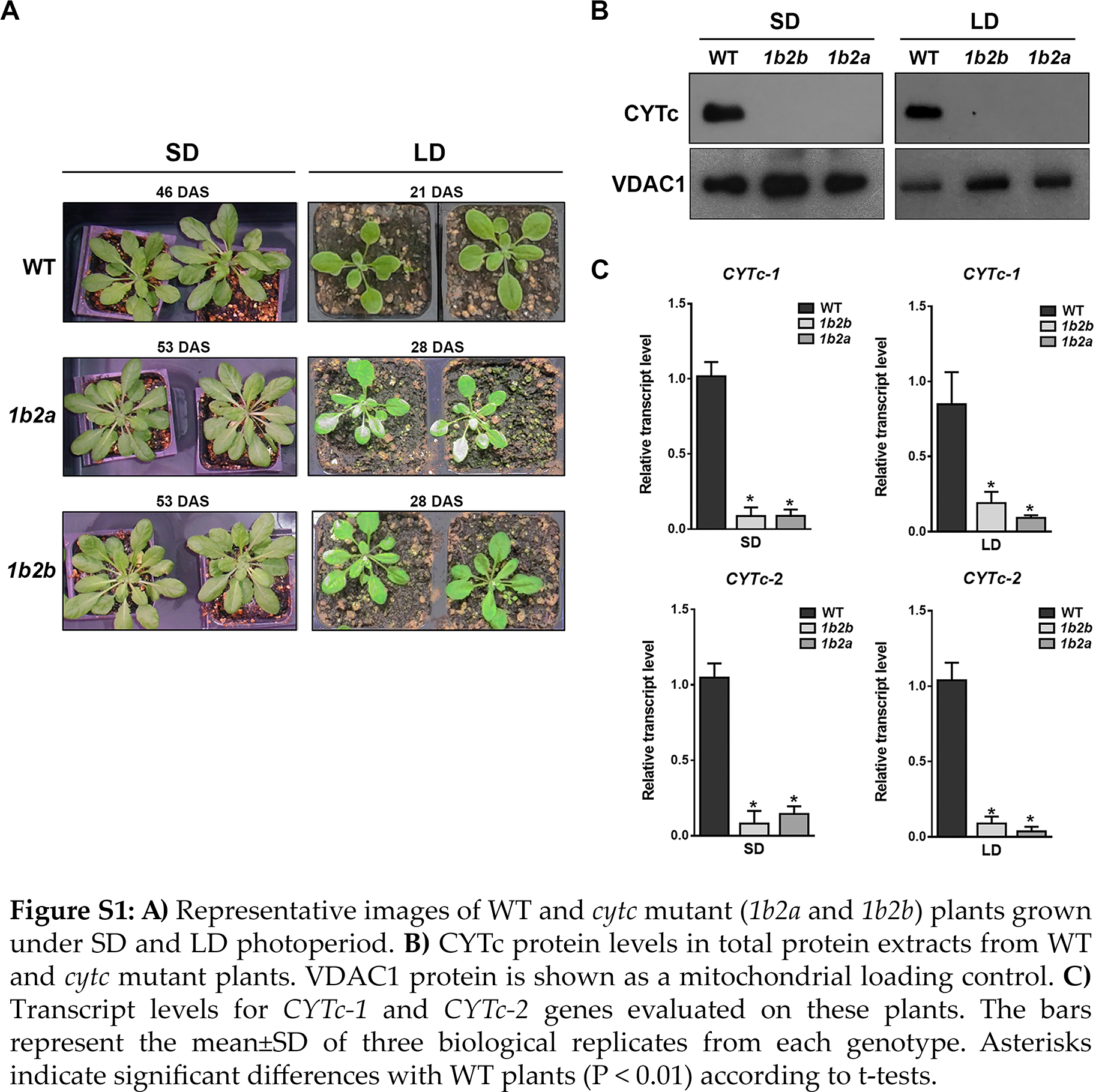

Supplement: Supplementary file 1 [file plants-10-00444-s001.zip › Florez-Sarasa et al_Plants_Supplementary Material/Figure S1.tif]

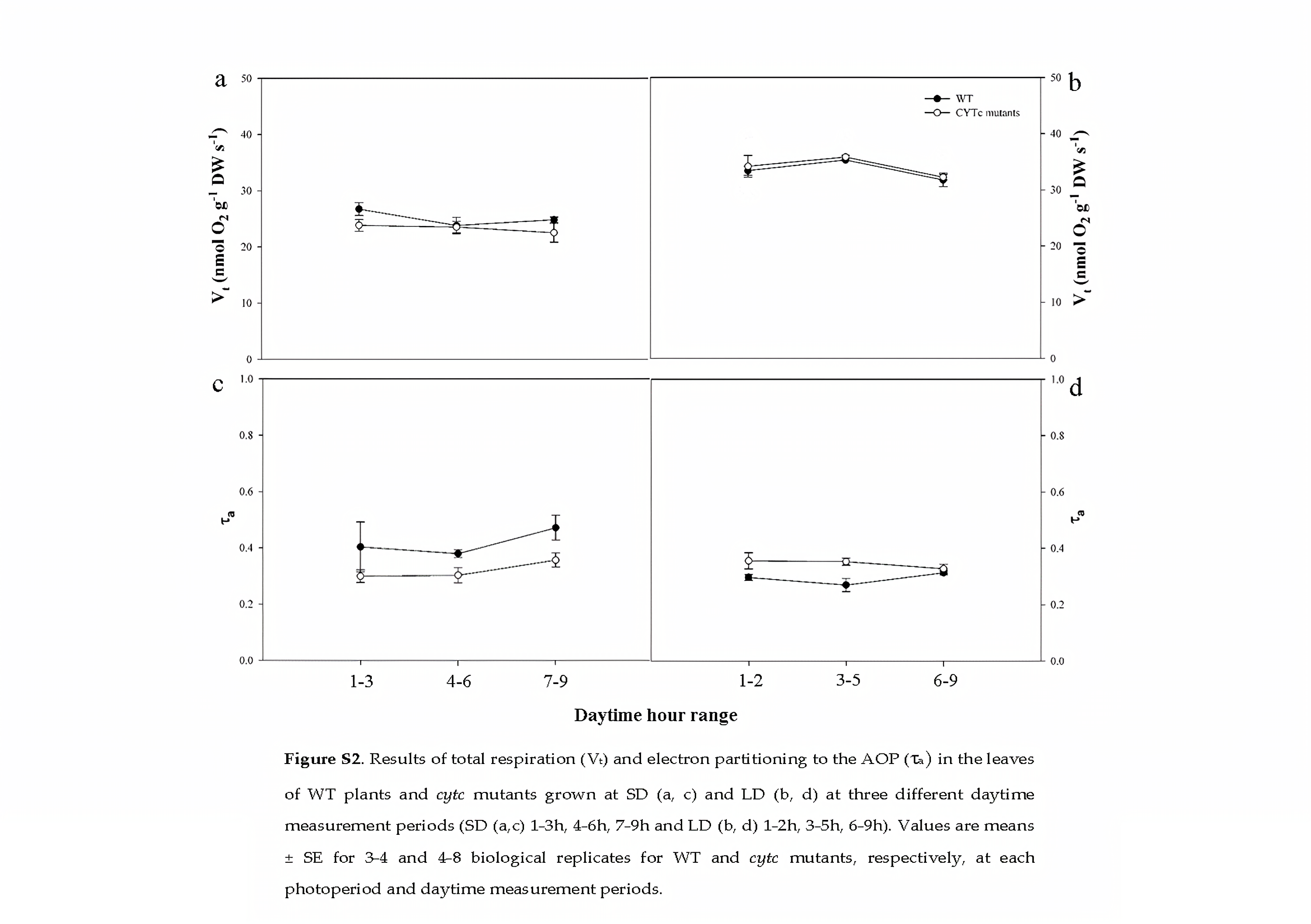

Supplement: Supplementary file 1 [file plants-10-00444-s001.zip › Florez-Sarasa et al_Plants_Supplementary Material/Figure S2.png]
